# The importance of social relationships in depression in autistic adolescents: a narrative-review

**DOI:** 10.3389/fpsyg.2024.1335882

**Published:** 2024-02-16

**Authors:** Élise Mamimoué, Christophe Gauld

**Affiliations:** ^1^Department of Child Psychiatry, Hospices Civiles de Lyon, Université de Lyon, Bron, France; ^2^Institut des Sciences Cognitives, UMR 5229 CNRS, Bron, France

**Keywords:** adolescent, assessment, autism spectrum disorder, major depressive disorder, social relationship

## Abstract

**Objective:**

The impact of social relationships on autistic adolescents has been recently studied. However, the link between social relationships and depression in autistic adolescents seem underrepresented in the scientific literature. Especially no specific assessment tool has been developed to evaluate depression in autistic adolescents. The aim of this narrative review is to raise the impact of social relationships on depression in autistic adolescents. We aim to propose lines of thought on the creation of a sensitive tool for identifying depression in this population.

**Methods:**

We conducted two types of searches for articles and reviews on PubMed, Embase, and Cochrane. First, regarding social relationships, we searched the following terms: [(“adolesc*” OR “youth”) AND (“ASD” OR “autis*”) AND (“social communication” OR “peer relationship”) AND (“depress*”)]. Secondly, regarding assessment tool, we searched the following terms: [(“tool” OR “assess*”) AND (“depress*”) AND (“ASD” OR “Autis*)”].

**Results:**

Social impact, verbal skills, and good social motivation are risk factors of depression in autistic adolescents. Social impairment during childhood is related to peer victimization and is a risk factor for depression. There is no specific tool to measure depression in autistic adolescents.

**Conclusion:**

No specific tool based on social relationships was developed to evaluate depression in autistic adolescents. Depression in autistic adolescents needs to be assessed considering the social and pragmatic specificities of autism. Social communication and difficulties in peer relationships may be evaluated in specific assessment tools based on social relationships for depression in autistic adolescents.

## Introduction

Autism is defined by impairments in communication and social interactions and restricted behaviors, interests, or activities (APA, 2013). Among autistic adolescents, 11–54% have major depressive disorder (hereinafter referred to as “depression”), and 13–79% have anxiety symptoms (Schwartzman and Corbett, 2020) and in the autistic pediatric population, depression rates variate from 0 to 83.3% (Stewart et al., 2022). Adolescence is a period at high risk of depression due to the characteristic hormonal and social changes as well as the increased academic challenges in this period (Hudson et al., 2019). However, in autistic adolescents, the mutual influences of peer relationships, depressive symptoms, and communication skills, weighted by autistic phenotype, seem difficult to determine.

Social communication difficulties is a core autistic symptom, and in autistic adolescents, social competence and quality of life are widely accepted to be improved by social skills groups (Reichow et al., 2013). Autistic behavioral phenotypes and the diversity of communication skills may be masked by depressive symptoms (Ghaziuddin et al., 2002; M. E. Stewart et al., 2006). Recognizing depressive symptoms in autistic adolescents, especially without language, is clinically difficult.

Several studies have evaluated the impact of social communication on the symptoms of depression in autistic young people as a part of their research. However, we did not identify any literature review specifically studying the impact of social relationships on depression in autistic adolescents. In the same way, we did not find any tool to assess depression in autistic adolescents while considering social communication and difficulties.

In this narrative review, we discuss the impact of social interaction on depression in autistic adolescents. Then, we highlight the lack of a specific diagnostic tool based on social communication for depression in this population.

## Materials and methods

This narrative review is based on scientific literature identified without time limit and until August 2023 and is conducted according to the Scale for the Assessment of Narrative Review Articles (SANRA) (Baethge et al., 2019). Our aims are: (1) to describe the link between depression and social relations for autistic adolescents; (2) to analyze studies that have been working on a tool to evaluate depression in youth with autim (3) to sustain the proposal of a specific tool assessing depression in autistic adolescents based on social relationships, and communication difficulties. We used two search strategies to identify relevant studies.

First, research databases PubMed, Embase, and Cochrane were conducted with the following terms in Title, Abstract and Keywords [(“adolesc*” OR “youth”) AND (“ASD” OR “autis*”) AND (“social com*” OR “peer relation*”) AND (“depress*”)]. Then, secondly, we analyzed articles that researched depression assessment tool in autistic adolescents. For the assessment part, we searched the following terms: [(“tool” OR “assess*”) AND (“depress*”) AND (“ASD” OR “Autis*)”]. We did this work separately, then regrouped our research and analyzed only the publications meeting the inclusion criteria. We considered review, communication, and original research. Duplicate records and research that did not focus on term words and aims were eliminated. We focused on articles that investigated the link between social communication, peer relationships and depression in autistic adolescents.

## Results

### Depression and social relationships in depression in autistic adolescents

In autism, mood disorder can have various forms of verbal and non-verbal expression. For instance, it can manifest as moral distress, impaired social communication, or even behavioral problems. The overlap between these clinical manifestations, specific to autistic adolescents with depressive symptoms, can be particularly difficult to untangle (Ghaziuddin et al., 2002; M. E. Stewart et al., 2006). As reported in the scientific literature (Muskett et al., 2019; Pezzimenti et al., 2019), the appearance of (or the increase in) repetitive or stereotypical behaviors, self-harm, and conduct difficulties incline clinicians to suspect depressive disorder in autistic adolescents with minimal verbal language. However, beyond autistic traits and genetic vulnerabilities of depression, several studies have shown increased depression among autistic adolescents.

In a non-systematic review, DeFilippis and collaborators (DeFilippis, 2018) pointed out the higher rate of depression in autistic children, relative to neurotypical people, and presented the impact of social communication impairment and negative life events on autistic youth with depression. In the same way, the finding of Pouw and collaborators shows that, unlike in neurotypical development individuals, negative friendships and victimization predicted depression in 52% of autistic adolescents (Pouw et al., 2013). This study also showed that individuals presenting avoidant strategies to manage stressful situations in the autistic group had fewer depressive symptoms. The authors considered these avoidant behaviors as adaptive coping strategies.

Hedley & Young (Hedley and Young, 2006) were the first to show correlation between social comparison and depression, especially perceived group membership in adolescent with autism. Rai and collaborators (Rai et al., 2018) showed that autistic adolescents who experienced social difficulties at age 7 and were bullied at age 10 had an increased risk of depression at age 18, relative to autistic adolescents, or neurotypical children who did not report these difficulties. The authors explained that social communication difficulties caused bullying and indirectly induced depression in autistic adolescents. These results were confirmed by a study of 176 autistic adolescents, which also found that social communication difficulties induced peer victimization and, thus, could lead to depression in this population (Greenlee et al., 2020).

Finally, the social impact of peers on the variability of expression of depressive symptoms in autism was further described in a review studying different factors (Pezzimenti et al., 2019): an average or high IQ, social motivation, and life stress such as bullying are risk factors of depression in autistic adolescent. The authors suggested that a good understanding of their social condition and their social particularities might induce depressive symptoms.

**Figure 1:**
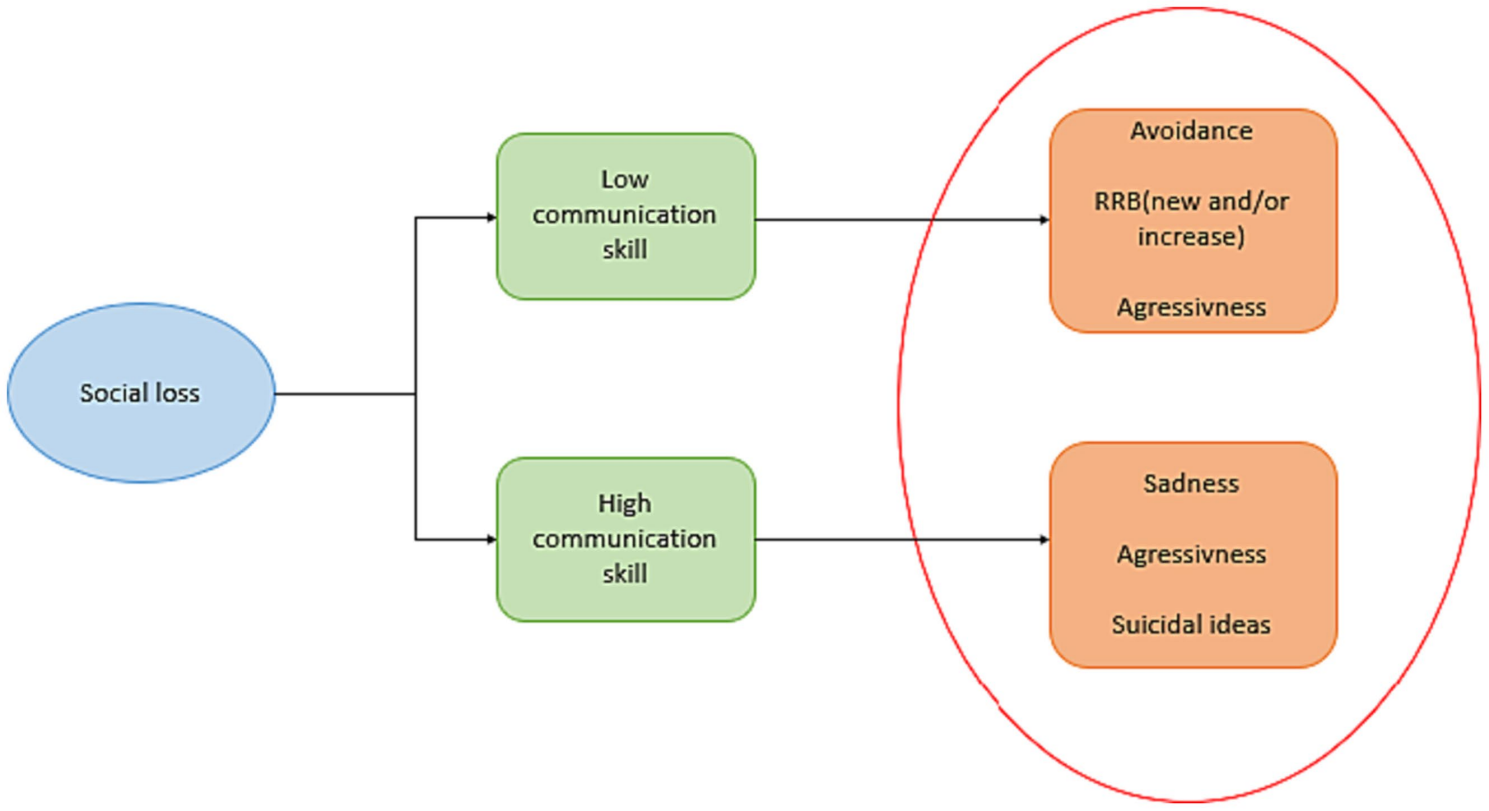
Impact of social loss on depressive symptoms of autistic adolescents according to their communication competence. There is a difference in depressive symptoms. Due to communication difficulties, autistic adolescents with communication difficulties express less sadness and suicidal ideas but show a behavioral response of avoidance or repetitive restrictive behavior (RRB) than autistic adolescents without communication difficulties. However (hetero- or auto-) aggressiveness manifests in both phenotypes.

Based on this scientific literature, we hypothesize that social environment and peer relationships, need to be considered in the diagnosis of depressive disorder in autistic adolescents. In this way, in Figure 1, we propose to exemplify the different influences of depression, behavioral phenotypes, peer relationships, and communication skills on autistic adolescents, taking the example of social loss as an environmental trigger of depression. We considered verbal language in the expression of depression, based on Greenlee et al. study. Greenle and collaborators (Greenlee et al., 2020) found that language impacts the difficulties of diagnosing depression in autistic adolescents. Autistic adolescents with verbal difficulties showed behavioral difficulties that may induce a lower rate of diagnosed depression in this population rather than the rate of behavioral difficulties.

### Finding a good assessment tool based on social items for depression in autistic adolescents

There is currently no specific tool based on social items for assessing depression among autistic adolescents. Indeed, standardized tools used on autistic adolescents focused on depressive symptoms were the Children’s Depression Rating Scale-Revised (CDRS-R) (Mayes et al., 2010), the Children’s Depression Inventory (CDI) (Kovacs, 2011), the Beck Depression Inventory (Kovacs, 2011), the Depression Anxiety Stress Scales (Lovibond and Lovibond, 1995), the Revised Child Anxiety and Depression Scale (RCADS) (Chorpita et al., 2000), the Short Mood and Feelings Questionnaire (SMFQ) (Sharp et al., 2006) and the criteria of the Diagnostic and Statistical Manual of Mental Disorders-5th edition (DSM-5) (APA, 2013). One of the major disadvantages of these scales is their lack of specificity regarding (adolescents with) ASD(Towbin et al., 2005).

A systematic review found only two studies that examined the psychometric properties of depression in autistic children, using two scales: the CDI and the RCADS (Kim, 2021). These assessment tools had lower sensitivity and specificity when used in autistic children relative to neurotypical children. In parallel, Ozsvadjian and collaborators proposed the Children’s Automatic Thoughts Scale (CATS) (Schniering and Rapee, 2002) for assessed depression in autistic young (Ozsivadjian et al., 2014).

To our knowledge, only three studies looked at the development of a specific scale for assessing depression in autistic adolescents. First, Leyfer et al. (2006) proposed the semi-structured Autism Comorbidity Interview–present and lifetime version (ACI-PL), based on the Kiddie Schedule for Affective Disorders and Schizophrenia (KSADS) (Kaufman et al., 1997). The authors screened responses to specify if individuals felt guilty, worthless, or how they understood separation anxiety. They also considered subsyndromal disorder due to the communication or cognitive impairment of some autistic adolescents. This study considered the point of view of autistic adolescents and, more precisely, how they phenomenologically understood their symptoms. KSADS did not evaluate communication impairment, peer relations impact, or bullying. Secondly, a pilot study using the PHQ-9 (for parents and as a self-questionnaire – validated in the TD population) evaluated depression in autistic youth (Pilunthanakul et al., 2021). The authors included youth aged 10–18 years diagnosed with autism and ensured the presence in their sample of children with depression. They used the Mini-Kids, a structured diagnostic interview for DSM-5 in children and adolescents (Sheehan et al., 2010), to evaluate “comorbid disorders.” Unfortunately, the study showed that the tool had lower sensitivity and specificity in autistic adolescents relative to the control population. A high level of depressive symptoms and suicidality was found in autistic adolescents. The authors also discussed the impact of social communication and victimization of autistic youth on their depressive symptoms, but they did not consider social communication and victimization for their assessment tool. Finally, a third study evaluated how the environment can predict depressive symptoms in autistic adolescents (Dallman et al., 2021). The authors used the CDI (self and parental report), the Positive and Negative Affect Scale (PANAS) Children’s Version, Short Form (Ebesutani et al., 2012) and the Ecological Momentary Assessment (EMA) (Shiffman et al., 2008)to evaluate depression in this population. The authors focused on momentary affects and trait factors on the population.

## Discussion

We have seen that a recent literature highlighted the impact of social relationships on the depressive symptoms of autistic adolescents (Pouw et al., 2013; DeFilippis, 2018; Rai et al., 2018; Greenlee et al., 2020). Loneliness and bullying are depressive symptom triggers related to social rejection, i.e., a loss of social ties (Pezzimenti et al., 2019), potentially impacting adult life (Lyznicki et al., 2004; Gray et al., 2012). The use of more sensitive and standardized psychometric tools for assessing depressive disorders in autistic adolescents could be of interest to clinicians and could give them a better view of the status of depression in autistic adolescents. However, none of these previous scales was based on communication and social interaction difficulties. Moreover, most of the assessing tools assessing depression in autism are self-report questionnaires. Autism being characterized by pragmatics difficulties, it is uncertain that autistic adolescents understand these self-questionnaires as well as the neurotypical population (Cassidy et al., 2021). This point shows the importance of an adapted tool for the autistic population w to assess depression based on social impairments.

The use of several scales aiming to evaluate social communication in austim, such as the Social Communication Questionnaire (Chandler et al., 2007), the Social Reciprocity Scale (Bruni, 2014), or the Children’s Communication Checklist (Bishop, 1998), could be of great interest. Social items seem to be important pillars in evaluating depression in autistic adolescents. However, to this date, none of these scales is adapted to depressive symptoms (Towbin et al., 2005).

Our study has limitations. First, the method of analysis and research follow the rules of a necessarily imperfect narrative review, which can lead to a risk of missing key references and induced biases in the analysis. However, we follow the systematic SANRA guidelines for this kind of review. Secondly, we found few studies with large samples (Lerner et al., 2018; Rai et al., 2018; Pilunthanakul et al., 2021), which may also induce a sample bias. Furthermore, most of the studies were not international, which did not allow to evaluate the impact of culture on the depressive symptoms. Thirdly, we only focused on the relation between peer relationships and their impact on depression in autistic adolescents. In this way, we are not exhaustive concerning global mental states, or therapies for depression in this population. Finally, we only considered scales that clearly diagnosed depression understood as a disorder (and not, for instance, anxiety, understood as a co-occurring).

## Conclusion and futures directions

Depression in autistic adolescents can be difficult to assess due to the complexity of the neurological difference, and also due to the lack of specific assessment tools based on the specific characteristics of this population (i.e., its different degrees of communication and social impairments). However, scientific literature shows the impact of communication and peer relationships on the depressive symptoms of autistic adolescents. Based on the absence of systematized scale built to assess the communicational and social impact on depressive symptoms in autistic adolescents, we propose in Table 1 five recommendations for future research to develop such a tool for this population, considering communication and peer relationships.

**Table 1:** Five recommendations to sustain the development of a specific assessment tool for depressive disorders in autistic adolescents based on communicational and social impacts.

**Explore the social environment of adolescents and especially peer relationships and bullying, in case of depressive symptoms**. Include the global state of the adolescent’s social environment (e.g., family and friends), the presence of rejection or bullying, and the quality of recent relationships. **Analyze behavioral change through the social relationships of autistic adolescents relative to their language**. Include the occurrence of self-injury, aggressiveness, and repetitive restrictive behavior. **Analyze the understanding of social and emotional items of the validated depression scales by autistic adolescents and compare their responses with non-autistic adolescents.** How autistic adolescents perceived peer relationships and their own emotions. Reformulate questionnaires with a better understanding of this population. **Evaluate the social skills and vocabulary of adolescents according to their language**. Language and pragmatics are variable in autism. The use of objective social and language assessment tools would allow the evaluation of the social and verbal skills of autistic adolescents. **Evaluate pleasure and interest in activities and specific interests.** Disinterest or loss of motivation in hobbies is a specific item of depression evaluation and should be a part of any systematic assessment.

## Author contributions

ÉM: Conceptualization, Investigation, Methodology, Resources, Validation, Writing – original draft, Writing – review & editing. CG: Investigation, Methodology, Resources, Supervision, Validation, Writing – review & editing.
